# Das Plaque‐type Trichoblastom und seine Geschwister: Eine (haarige) Familiengeschichte: Plaque‐type trichoblastoma and its siblings: A (follicular tumour) family history

**DOI:** 10.1111/ddg.70255x

**Published:** 2026-11-10

**Authors:** Ines Bertlich, Ferdinand Toberer, Tilman Schulz, Dmitry V. Kazakov, Wolfgang Hartschuh

**Affiliations:** ^1^ Universitätsklinikum Heidelberg Abteilung für Dermatologie Heidelberg Deutschland; ^2^ Institut für Pathologie Klinikum Bayreuth GmbH Bayreuth Deutschland; ^3^ IDP Institut für Dermatohistopathologie Pathologie Institut Enge Zürich Schweiz; ^4^ Hautarztpraxis Durani Heidelberg Deutschland

Sehr geehrte Herausgeber,

 eine 61‐jährige Patientin stellte sich aufgrund einer seit zwei Jahren bestehenden Plaque in der linken Nasolabialfalte vor, welche in den vergangenen sechs Monaten begonnen hatte zu jucken und zu wachsen. Die klinische Untersuchung zeigte eine unscharf begrenzte, hautfarbene Plaque von etwa 4 mm Durchmesser (Abbildung 1a–c). Es wurde eine optische Kohärenztomographie (OCT) durchgeführt. Dabei zeigte sich ein ausgedehnter, kleinknotiger Tumor, der die untere Bildgrenze des OCT‐Bildes überschritt (Abbildung 1d).

**ABBILDUNG 1:**
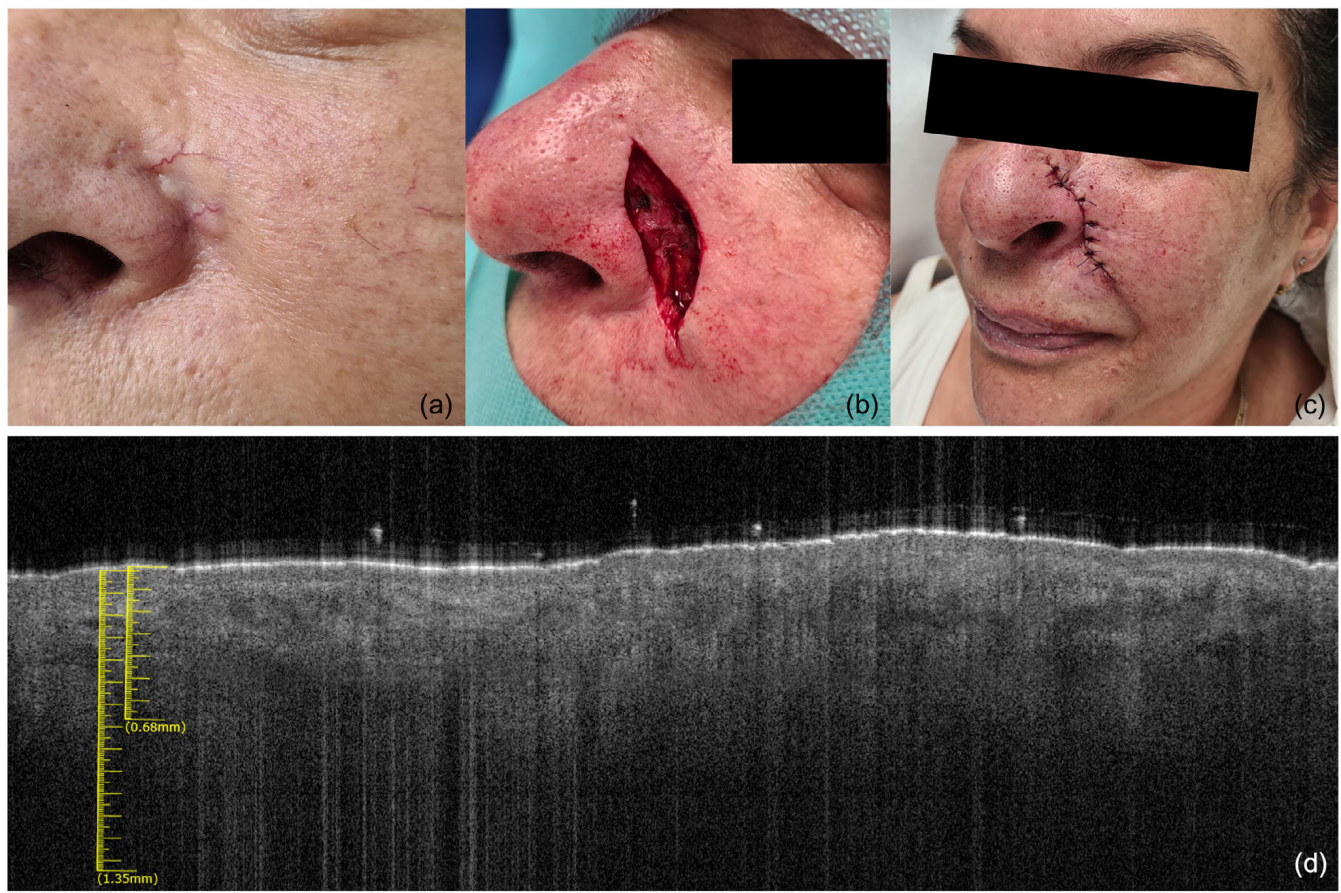
(a) Erstvorstellung der Patientin mit einer hautfarbenen Plaque und prominenten Teleangiektasien; (b) Intraoperativ; (c) Direkt postoperativ; (d) OCT‐Bild mit Darstellung eines ausgedehnten, kleinknotigen Tumors. Die basale Tumorgrenze ist im OCT‐Bild nicht darstellbar.

Eine Spindelbiopsie zeigte einen basaloiden, kleinknotigen Tumor mit reichlich dichtem, zellreichem Stroma ohne epidermale Verbindung (Abbildung 2a–c). Es fanden sich Mitosen sowie eine diskrete periphere Palisadenstellung der Tumorzellen. Innerhalb einiger Tumorknoten waren runde Areale mit einer helleren Zellpopulation sichtbar („ball‐like structures“, Abbildung 1c).^1^ Infiltratives Wachstum der Tumorknoten in oberflächliche mimische Muskelschichten war erkennbar (Abbildung 2b). Die immunhistochemische Untersuchung zeigte eine starke Positivität für BerEP4 und AE1/3, eine deutlich erhöhte Proliferationsaktivität in der Ki‐67‐Färbung sowie bemerkenswerterweise eine erhöhte Dichte von CK20‐positiven Merkel‐Zellen in einigen der Tumorknoten (Abbildung 2d–f).

**ABBILDUNG 2:**
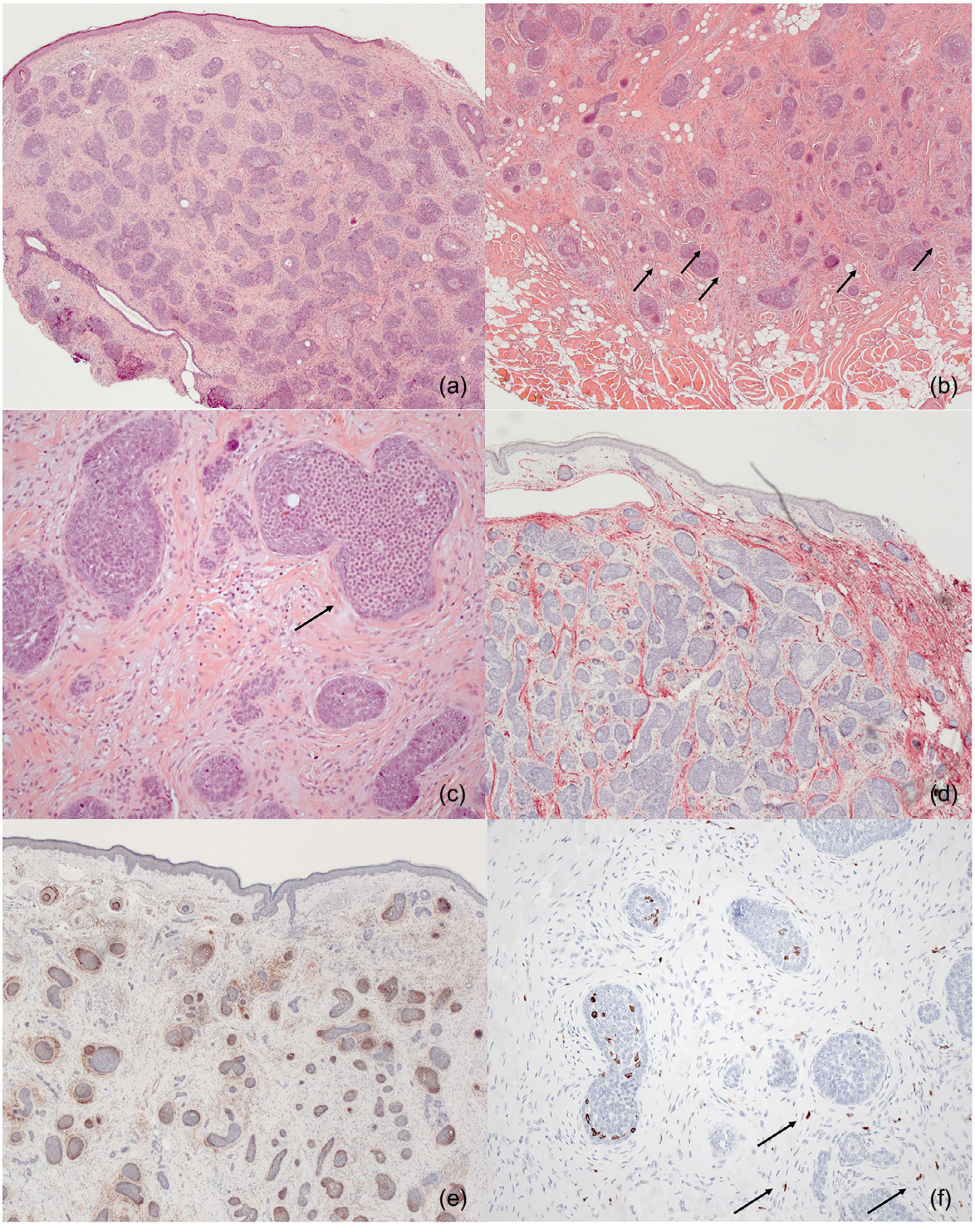
(a) HE‐Färbung, 25‐fache Vergrößerung: Kleine basaloide Tumorknoten ohne epidermale Verbindung und mit ausgedehntem, zellreichem Tumorstroma. (b) HE‐Färbung, 25‐fache Vergrößerung: Infiltratives Wachstum in mimische Muskulatur (Pfeile) und subkutanes Fettgewebe. (c) HE‐Färbung, 100‐fache Vergrößerung: Ball‐like structures (Pfeil). (d) CD34‐Färbung, 25‐fache Vergrößerung, roter Chromogen: Diffus positiv im Tumorstroma und im lokalen Bindegewebe. (e) CD10‐Färbung, 25‐fache Vergrößerung, brauner Chromogen: Diffus positiv in den Tumorknoten und im Tumorstroma. (f) CK20‐Färbung, 100‐fache Vergrößerung, brauner Chromogen: Einzelne Tumorknoten mit vermehrten CK20‐positiven Merkelzellen innerhalb der Knoten und im peritumoralen Stroma (Pfeile), ein typischer Befund der perifollikulären Bulge‐Region.

Wir diagnostizierten ein Plaque‐type Trichoblastom, eine seltene, infiltrativ wachsende Variante des Trichoblastoms.

Zu den klinischen und histologischen Differentialdiagnosen zählen andere follikuläre Tumoren wie das Basalzellkarzinom (BCC) und das „konventionelle“ Trichoblastom (TB). Diese Tumorentitäten gelten als eng miteinander verwandt und zeigen eine Differenzierung in Richtung der Bulbus‐Papillen‐Region des Haarfollikels, sodass sie eine „follikuläre Tumorfamilie“ bilden.^2^ Während beim BCC eine Differenzierung nur in Richtung follikulärer Bulbuszellen angenommen wird, differenziert das TB sowohl in Richtung follikulärer Bulbuszellen als auch in Richtung follikulärer Papillenzellen.^2^ Dies erklärt, warum das BCC nur ein eher spärliches Tumorstroma aufweist und seine Retraktionsartefakte (Clefts) zwischen den Tumorknoten und dem Tumorstroma liegen, wohingegen das TB reichlich zellreiches Tumorstroma besitzt und die Spalten zwischen dem Tumorstroma und dem umgebenden lokalen Bindegewebe lokalisiert sind. Zusätzlich fehlt dem TB eine epidermale Verbindung, die beim BCC häufig vorhanden ist, und es kann papillary mesenchymal bodies zeigen: zellreiche bindegewebige Knoten, die der Haarfollikelpapille ähneln und die von den Tumorzellen klauenartig umgriffen werden (in unserem Fall nicht sichtbar).

Allerdings gibt es in der Literatur auch TB‐Fälle mit BCC‐ähnlichen Arealen,^3^ sowie auch Berichte über ein Trichoblastom, dessen Diagnose später zu einem trichoblastischen Basalzellkarzinom revidiert wurde.^4^, ^5^ Dies zeigt, dass zwischen diesen Entitäten eine erhebliche Überlappung besteht.

Das Plaque‐type Trichoblastom weist einige histologische Merkmale auf, die stark an ein Basalzellkarzinom erinnern, wie etwa ein lokal infiltratives Wachstum sowie ein unscharf begrenztes, plaqueförmiges klinisches Erscheinungsbild. Gleichzeitig teilt es aber auch Eigenschaften mit dem TB, beispielsweise das zellreiche, weitläufige Stroma. In der Literatur wird sein biologisches Verhalten als gutartig bis niedrig‐maligne mit lokaler Infiltration eingeschätzt, jedoch ohne Berichte über Fernmetastasen.^4^, ^6^, ^7^, ^8^ Auch bei unserer Patientin waren zwei Exzisionen notwendig, um tumorfreie Schnittränder zu erreichen.

Die histologische Diagnosestellung basiert überwiegend auf der HE‐morphologischen Untersuchung (Tabelle 1). Obwohl zahlreiche verschiedene immunhistochemische Färbungen zur Unterstützung der Unterscheidung dieser Entitäten vorgeschlagen wurden, besitzen die meisten davon nur eine begrenzte diskriminatorische Aussagekraft (Online‐Supplement Tabelle S1). Die CK20‐Expression scheint die hilfreichste immunhistochemische Analyse zur Unterscheidung zwischen BCC und „konventionellem“ TB zu sein: Während beim TB erhöhte Zahlen CK20‐positiver Merkel‐Zellen innerhalb der Tumorknoten auftreten können, ist dies beim Basalzellkarzinom nicht der Fall.^9^ Nach unserem Kenntnisstand ist dies der erste Bericht zur CK20‐Expression beim Plaque‐type Trichoblastom. In diesem Fall zeigte sich fokal eine erhöhte Anzahl CK20‐positiver Zellen in einigen der Tumorknoten, analog zum „konventionellen“ TB.

**TABELLE 1:** Vergleich von BCC, Plaque‐type Trichoblastom and “konventionellem“ Trichoblastom.

		BCC	Plaque‐type Trichoblastom	Trichoblastom
**H&E Merkmale**	**Architektur**	Schlecht umschrieben	Schlecht umschrieben	Gut umschrieben
	**Kleine basophile Tumorknoten**	Ja	Ja	Ja
	**Periphere Palisadenstellung der Tumorzellen**	Ja	Ja	Ja
	**Tumor Stroma**	Spärlich	Zellreich, reichlich	Zellreich, moderat
	**Invasives Wachstum**	Ja	Ja	Nein
	**Tumor ist leicht aus dem Bindegewebe herauszulösen**	Nein	Nein	Ja
	**Clefts**	Zwischen Tumorknoten und Stroma	Nein ^10^	Zwischen Tumorstroma und umgebendem Bindegewebe
	**Epidermisverbindung**	Ja	Nein	Nein ^7^
	**Ball‐like structures**	Nein	Ja (im vorliegenden Fall sichtbar) Keine weiterführenden Daten vorhanden	Nein
	**Papillary mesenchymal bodies**	Nein	Ja	Ja

In einzelnen Fällen wurden somatische Mutationen im PTCH1‐Gen von Plaque‐type Trichoblastomen nachgewiesen, einem Tumorsuppressorgen und Bestandteil des Sonic‐Hedgehog‐Signalwegs. PTCH1‐Mutationen sind ein charakteristischer Befund beim Basalzellkarzinom sowie beim Goltz‐Gorlin‐Syndrom.^6^, ^10^ In unserem Fall konnten wir jedoch eine Mutation im PTCH1‐Gen ausschließen.

Zusammenfassend handelt es sich beim Plaque‐type Trichoblastom um einen seltenen, lokal infiltrativ wachsenden Tumor mit Differenzierung in Richtung von Haarfollikelbulbus und ‐papille, der gemeinsam mit dem Basalzellkarzinom und dem „konventionellen“ TB eine „Tumorfamilie“ innerhalb der Haarfollikeltumoren bildet. Aufgrund ihrer engen Verwandtschaft ist die Abgrenzung dieser Tumoren klinisch wie auch histologisch schwierig. Unser Fall zeigt, dass auch das Plaque‐type Trichoblastom, ähnlich dem „konventionellen“ TB, fokal vermehrt CK20‐positive Merkel‐Zellen aufweist.

Aufgrund der großen Bedeutung architektonischer und morphologischer Kriterien für die Diagnosestellung sollten Exzisionsbiopsien gegenüber Shave‐Biopsien klar bevorzugt werden. Wir sehen für die optische Kohärenztomographie (OCT) aufgrund ihrer im Vergleich zur Tumorinfiltrationstiefe begrenzten Eindringtiefe keinen relevanten diagnostischen Vorteil. Aufgrund des lokal infiltrativen Wachstumsmusters empfehlen wir eine vollständige Exzision.

## DANKSAGUNG

Open access Veröffentlichung ermöglicht und organisiert durch Projekt DEAL.

## INTERESSENKONFLIKT

Keiner.

## Supporting information



Supporting Information
