# Supplementary material for: Das Plaque‐type Trichoblastom und seine Geschwister: Eine (haarige) Familiengeschichte: Plaque‐type trichoblastoma and its siblings: A (follicular tumour) family history
Source: J Dtsch Dermatol Ges. 2026 Nov 10;24(11):1643–6. [Article in German] doi: 10.1111/ddg.70255x (PMC13749584; doi:10.1111/ddg.70255x)
Supplement: Supplementary file 1 [file DDG-24-1643-s001.docx]

**Supplement Tabelle S1:**

Immunhistochemischer Vergleich von BCC, Plaque-type Trichoblastom und „konventionellem“ Trichoblastom

|  |  | **BCC** | **Plaque-type Trichoblastom** | **Trichoblastom** |
| --- | --- | --- | --- | --- |
| **Immunhisto-chemische Merkmale** | **BerEP4** | Positiv in Tumorknoten  ^7^ | Positiv in Tumorknoten  ^4^  Negativ oder fleckförmig positiv  ^7^ | Positiv in Tumorknoten  ^7^ |
|  | **CK20** | Keine positiven Merkelzellen in Tumorknoten  ^9^ | Fokal erhöhte CK20+ Merkelzellen in Tumorknoten (im vorliegendene Fall sichtbar)  Keine weiterführenden Daten vorhanden | Vermehrte positive Merkelzellen in Tumorknoten  ^9^ |
|  | **CD10** | Positiv in Tumorknoten, negativ in Tumorstroma  ^7^ | Diffus positiv in Tumorstroma und umgebendem Stroma  ^10^ und ^7^ | Positiv in Tumorstroma  ^7^ |
|  | **CD34** | Negativ in Tumorknoten und in Tumorstroma  ^7^  Positiv in Tumorstroma  ^4^ | Diffus positiv in Tumorstroma und umgebendem Stroma  ^7^  Positiv in Tumorstroma  ^4^^,^^10^ | Positiv in Tumorstroma  ^7^ |
|  | **Bcl-2** | Diffus positiv  ^7^ und ^4^ | Positiv in direktem Tumorstroma  ^7^  Diffus positiv in Tumorknoten  ^4^ | Positiv in peripheren Tumorknoten  ^7^ und ^4^ |
|  | **PHLDA1** | Negativ  ^8^ | Variables Ausmaß der Positivität  ^8^ | Positiv ^11^ |

Orangefarbene Flächen= Fälder, in denen die Befunde verschiedener Autoren nicht übereinstimmen

Tumorstroma = Stroma, das Teil des Tumors ist und direkt an die Tumorknoten angrenzt bzw. diese umgibt

Umgebendes Stroma = nicht-tumoröses, gesundes Stroma, in das Tumorknoten und Tumorstroma eingebettet sind
